# Effect of perioperative forced air warming blanket on older adult patients undergoing spinal surgery with general anesthesia

**DOI:** 10.12669/pjms.40.4.7886

**Published:** 2024

**Authors:** Youngduck Shin, Jooyong Lee, Haewon Seo, Sanghi Park

**Affiliations:** 1 Youngduck Shin, Department of Anesthesiology and Pain Medicine, Chungbuk National University College of Medicine, Cheongju, Republic of Korea. Department of Anesthesiology and Pain Medicine, Chungbuk National University Hospital, Cheongju, Republic of Korea; 2 Jooyong Lee, Department of Anesthesiology and Pain Medicine, Chungbuk National University Hospital, Cheongju, Republic of Korea; 3 Haewon Seo, Department of Anesthesiology and Pain Medicine, Chungbuk National University Hospital, Cheongju, Republic of Korea; 4 Sanghi Park, Department of Anesthesiology and Pain Medicine, Chungbuk National University Hospital, Cheongju, Republic of Korea

**Keywords:** Anesthesia, General, Hypothermia, Perioperative Care

## Abstract

**Objective:**

As the population of patients aged 65 years and older increases, the number of older adult patients undergoing surgery also increases. Older adults are vulnerable to hypothermia due to age-related changes in the thermoregulatory center, which leads to reduced subcutaneous fat tissue, vasomotor response, and heat production. Thus, they are more likely to suffer complications, including cardiovascular changes, blood coagulation disorders, infections, and delayed recovery from surgery. The study investigated the effect of preventive active warming.

**Methods:**

This retrospective cohort study conducted at Chungbuk National University Hospital investigated clinical data from older adult patients undergoing spinal surgery from January 1, 2020, to December 13, 2022. In this study, we explored the use of prophylactic active warming during anesthesia induction and post-surgery warming in older adult patients (≥65 years) who experienced hypothermia during and after surgery under general anesthesia.

**Results:**

The control group of patients who experienced hypothermia increased from 20% after 10 minutes to 80% after 30 minutes and 100% after 60 minutes. The percentage of patients in the treatment group who initially experienced hypothermia increased from 10% after 30 min to 40% after 60 minutes. However, notably, 90% of these patients had returned to a normal body temperature upon their arrival at the recovery room. The difference in the percentage of patients who developed hypothermia was statistically significant between the two groups.

**Conclusions:**

Hypothermia prevention via an air-forced warming blanket was effective for older adult patients undergoing spinal surgery under general anesthesia.

## INTRODUCTION

As the population of patients aged 60 years and older increases, the number of patients requiring urological and orthopedic surgery also increases. Subsequently, there has been a rise in spinal surgeries performed on older adult patients, and this requires general anesthesia. It is important to consider that the core body temperature of older adult patients decreases faster than that of younger adults under general anesthesia. Moreover, older adult patients require a more extended recovery period.^1^ Therefore, the risk of hypothermia is considered. Hypothermia is defined as a core temperature of less than 36.0°C in the tympanic membrane, nasopharynx, or rectum.^2^

Hypothermia affects 70% of patients under general anesthesia.^2^ General anesthesia, low temperature environment in the operating room, and the intravenous and lavage fluids used during surgery cause body temperature control disorders.^3^ Decreased body heat production and thermoregulatory vasoconstriction due to aging increase the risk of intraoperative hypothermia. The heat loss that occurs under general anesthesia is due to the altered thermoregulatory functions caused by the anesthesia.

Specifically, it interferes with shivering and peripheral blood vessel dilatation. Patients undergoing spinal surgery in the prone position may be susceptible to hypothermia given the extended duration of the procedure and the substantial skin surface exposed during surgery.^4^ Older adult patients typically have concomitant cardiovascular diseases. Thus, the risk of complications, such as cardiovascular abnormalities, blood coagulation disorders, infections, and delayed recovery, is increased due to intraoperative hypothermia.^3^

Although there are studies on intraoperative hypothermia in older adult patients, there are few studies that have confirmed the efficacy of preventive active warming during surgeries where the possibility of hypothermia is high, such as spinal surgery. This study investigated the use of prophylactic active warming during anesthesia induction in older adult patients under general anesthesia during spinal surgery.

## METHODS

Clinical data of 20 older adult patients who underwent spinal surgery at our institution from January 1, 2020, to December 13, 2022, were retrospectively analyzed. The study included older adult patients (65 years and older), who underwent spinal surgery under general anesthesia with an anesthesiologist grade I-II (American Society of Anesthesiologists Physical Grade (ASA) of I–III). Patients with unstable vital signs were excluded. Prior to general anesthesia, no premedication was administered, and the patients were transferred to the operating room with standard monitoring. Inhaled anesthesia, consisting of sevoflurane, was administered, and anesthesia was induced with propofol, 2–3 mg/kg IV, rocuronium 0.6–1.2 mg/kg, and remifentanil 0.25–0.5 mcg/kg/minutes.

### Ethical Approval

This study was approved by the Institutional Review Board of Chungbuk National University Hospital (IRB approval no. CBNUH 2022-11-020-001).

Ten patients received active warming with an air-forced warming blanket (Bair Hugger, 3 M, Maplewood, MN, USA) while anesthesia was induced. Another 10 patients in the control group did not receive active warming. The two groups were compared by recording body temperature before anesthesia induction, 10, 30, and 60 min after anesthesia induction, upon arrival in the recovery room or intensive care unit, and 20 min after arrival in the recovery room or intensive care unit.

Temperature measurements were recorded at the tympanic membrane using the same thermometer each time to maintain consistency and reduce errors. (Thermoscan IRT4520, infrared thermometer, Braun, Germany). All patients had a blood warmer in use while on the bed. A humidified circuit was used for intraoperative transfusions or rapid intravenous infusions.

Before the end of the surgery, analgesia was administered using intravenous patient-controlled analgesia (IV PCA). Any shivering was noted in the postoperative recovery room or intensive care unit during routine post-operative observations.

In a previous pilot experiment, when an active forced warming blanket was used from the beginning, the incidence of hypothermia in patients over 65 years old was 10.2%. However, when the warming blanket was not used, the incidence of hypothermia rose significantly to 63.9%. The results revealed a stark difference, and recognizing the importance of these findings, we then performed sample size calculations. After performing G power calculations, assuming a statistical power of 80% (α = 0.05), we determined that a minimum sample size of 18 individuals per group was necessary. Accounting for a dropout rate of 10%, the final sample size was set at 10 patients per group. Statistical analysis was performed using SPSS for Windows software (version 27.0; IBM Corp., Armonk, NY, USA). The two groups were compared using the Student’s *t* test for continuous data. Shapiro-Wilk test was used to confirm normality, and the chi-square test was used to evaluate categorical data. Descriptive statistics were expressed as the mean ± standard deviation, median (minimum–maximum), frequency distribution, and percentage. Statistical significance was established at *P* < .05.

## RESULTS

No statistically significant differences were observed between the two groups in terms of sex, height, weight, ASA grade of the anesthesiologist, surgical technique, and anesthetic time. (Table-I). The values presented here denote either patient counts or mean values accompanied by their respective standard deviations. No statistically significant differences were identified when comparing the two groups.

**Table-I:** Demographic data of patients.

	Active warming (n=10)	Control Group (n=10)
Sex ratio (M/F)	3/7	3/7
Age (year)	73.40 ± 6.69	76.20 ± 6.63
Height (cm)	153.93 ± 8.76	155.96 ± 10.86
Weight (kg)	57.86 ± 9.80	58.92 ± 12.30
ASA(I/II/III)	1/4/5	1/5/4
Anesthetic time (min)	262.00 ± 84.86	231.00 ± 67.40
Operative time (min)	219.50 ± 81.56	192.50 ± 67.34
OR ambient Temp (0C)	21.19 ± 0.17	21.28 ± 0.19
PACU ambient Temp (0C)	24.96 ± 0.12	25.01 ± 0.14

**Fig.1:**
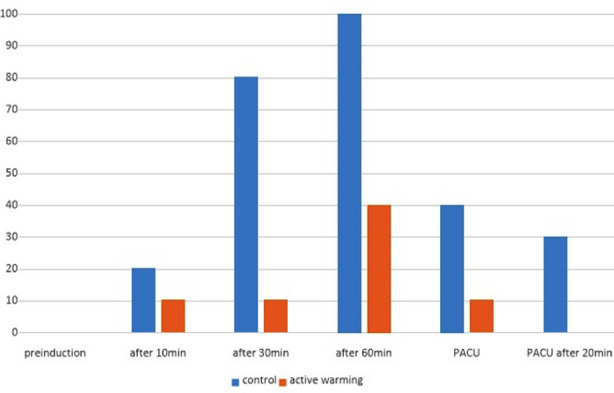
Hypothermia rate.

The percentage of patients in the treatment group who initially experienced hypothermia increased from 10% after 30 minutes to 40% after 60 minutes. However, it is worth noting that 90% of these patients had returned to a normal body temperature upon their arrival at the recovery room. (Fig.1). The percentage of patients who developed hypothermia was significantly lower in the treatment group at 10, 30, and 60 minutes after anesthesia, as well as upon arrival at the recovery room. The difference in the rates of hypothermia was also statistically significant between the two groups (Table-II). Shivering was not observed in any of the patients in either group. (Fig.1.) The percentage of patients in the control group who developed hypothermia increased from 20% after 10 minutes to 80% after 30 min and 100% after 60 minutes.

**Table-II:** Hemodynamics monitoring in the two groups.

	Active warming (n=10)	Control Group (n=10)	P
Pre-induction (^0^C)	36.42 ± 0.36	36.26 ±0.16	.21
after 10 mins (^0^C)	36.36± 0.33	36.08±0.17*	.029
after 30 mins (^0^C)	36.27 ±0.32	35.76 ± 0.16*	.000
after 60 mins (^0^C)	36.08± 0.28	35.61 ± 0.16*	.000
PACU (^0^C)	36.56±0.45	36.02± 0.48*	.018
PACU after 20 mins (^0^C)	36.52 ± 0.34	36.18 ± 0.51	.096

Values represent the number of patients and all continous data are presented as mean ± standard deviation. Pre-induction, just before anesthesia induction; after 10 mins., 10 mins. after the initiation of induction; after 30 minutes, 30 minutes after the initiation of induction; after 60 minutes., 60 minutes. after the initiation of induction; PACU, after arriving in the PACU; PACU after 20 minutes, 20 minutes. after arriving in the PACU. −*P* < .05 vs group C.* indicates *P* < .05.

## DISCUSSION

In this study, we examined the impact of using an air-forced warming blanket for prophylactic hypothermia prevention in older adult patients vulnerable to surgical hypothermia, specifically before vasodilation occurs due to anesthesia induction. In spinal surgery, the patient is placed prone and securely immobilized following anesthesia. The surgical site is thoroughly disinfected, and the C-arm X-ray machine is used for imaging purposes. The preoperative preparation takes an extended time due to the thorough evaluation of the surgical site. In this study, maintaining postoperative body temperature through air-forced warming was challenging. Consequently, some patients were divided into three groups: first group received pre-installed and heated air-forced warming blankets; second group did not have prior installation and experienced intraoperative hypothermia, and the third group received warm air beneath surgical cover.

The body temperature of the older adult patients was monitored in anticipation of hypothermia development. The body temperature of those patients who did not undergo active warming under general anesthesia began to decrease after anesthesia induction. The percentage of patients in this group who developed hypothermia increased from 20% after 10 min to 60% after 60 min. The difference in the percentage of patients who developed hypothermia was statistically significant from 10 min after anesthesia induction to their arrival at the recovery room.

In surgical patients, the skin temperature increases due to the vasodilatory effect of anesthesia. However, the risk of hypothermia increases due to the cooling effect of disinfection, heat loss at the surgical site, and reduced metabolism due to anesthesia. Major and prolonged operations can cause hypothermia due to increased heat loss.^5^ The recommended average operating room temperature is 20–23 °C, and the average operating room and recovery room temperature at our hospital was 21.19 ± 0.17 °C and 21.28 ± 0.19 °C. A low temperature in the operating room further promotes patient heat loss. Patients with advanced ages (≥65 years), low preoperative body temperatures, a combination of local and general anesthesia, prolonged operation times, large incisions, heavy blood losses and requiring fluid replacement as well as an operating room temperature of less than 21°C, are all risk factors for hypothermia.^6^-^8^

Moreover, patients with a BMI of 25 kg/m^2^ or higher and those with chronic diseases have an increased risk for hypothermia.^9^ In 2020, the population aged 65 years or older in Korea was 8,123,432, which accounted for 15.7% of the total population.^10^ As the number of patients aged 65 years or older increases, the number of older adult patients undergoing surgery also increases. Age-related changes in the thermoregulatory center were attributed to reduced subcutaneous adipose tissue, a reduced vasomotor response, and an inability to generate body heat for various physiological reasons. Older adult patients often present with concomitant cardiovascular diseases. Therefore, they are at risk of complications, including cardiovascular changes, blood coagulation disorders, infection, and delayed surgical recovery due to intraoperative hypothermia.

Older adult patients are more vulnerable to drug interactions and side effects compared to younger patients, and age-related chronic diseases increase the possibility of complications for various drugs. Specifically, the perioperative mortality rate increased by three to five times. Another issue among older adult patients experiencing hypothermia is shivering, which significantly increased basal metabolic rate. Failure to compensate for increased oxygen consumption results in arterial hypoxemia. Additionally, the cardiac output increases secondary to peripheral vasoconstriction during shivering. This response leads to myocardial ischemia due to the increased cardiac burden. Many drugs and gases used for general anesthesia alter the thermoregulatory response. Compared with younger patients, postoperative hypothermia is more likely to occur and last longer in older adult patients due to the reduced clearance rate of the anesthetics.^11^-^13^

Previously reported methods for preventing hypothermia include intravenous fluids, blood products, and intraoperatively heated lavage fluids.^14^ Based on the results of a previous meta-analysis, the active heating technique, using an air-forced warming blanket, was more effective in preventing hypothermia than the passive method of covering the patient with mats or covers.^15^

Hypothermia poses a risk of myocardial ischemia, wound infection, coagulopathy, and drug metabolism disorders. In patients with reduced cardiac and lung functional reserves, shivering worsens their condition due to the significantly increased metabolic stress.^3^,^16^-^17^ As the number of older patients is rising, establishing effective methods, such as proactive warming and body temperature monitoring, to maintain hemodynamic stability among patients undergoing spinal surgery is essential.

Forced-air warming is the most effective method to prevent hypothermia in patients during surgery. Research on the effects of forced air warming is underway in various fields, such as the effects of differences in pre-surgical warm-up time, the effects of upper and lower body blankets, and the effectiveness of blankets in urological and abdominal surgeries.^18^-^23^ Although the effectiveness and optimal timing of prewarming are controversial, prewarming is important for vasodilation that occurs before and after anesthesia induction, especially in older adult patients vulnerable to hypothermia. Spinal surgery involves considerable exposure of the surgical area, changes in position, and extended surgical times. Therefore, 63.9% of older adult patients who were not prewarmed in the preliminary survey showed hypothermia. Studies on intraoperative hypothermia in older adult patients have been conducted;^24^,^25^ however, few studies have confirmed the effectiveness of prophylactic active warming in spinal surgery. In this study, we confirmed that continuous warming along with prewarming is essential for maintaining perioperative body temperature in older adult patients.

### Limitations

First, it concerns the methodology of using a forced air-warming blanket. A study showed that warming the upper body in a supine position is more effective than warming the lower body;^18^,^19^ however, the effect of warming the upper and lower body in older adult patients in a prone position has not been confirmed. Buraimoh et al. reported there was no difference in the insulation effectiveness between the upper and lower body blankets in patients who underwent spinal surgery,^20^ but there were also results showing that the upper body blanket was more effective.^21^ Therefore, further studies are needed to identify separate patient groups. Second, the effect of forced air warming at start time could not be confirmed. It is necessary to understand the effect of prophylactic air warming more accurately by dividing the patient group into three groups: after the start of anesthesia, after the start of surgery, and after the start of the onset of hypothermia. Lastly, since this study is a single-center, small-sample study, it is necessary to confirm the results through a large-sample multicenter study.

## CONCLUSION

Intraoperative core body temperature monitoring and maintenance via an air-forced warming blanket were essential to prevent hypothermia among older adult patients undergoing spinal surgery under general anesthesia.

### Authors’ Contributions:

**YS and SP:** Conceived and designed the study.

**YS, JL, HS, and SP:** Collected the data and performed the analysis.

**YS and SP:** Was involved in the writing of the manuscript and is responsible for the accuracy and integrity of the study.

All authors have read and approved the final manuscript.
